# Lynx1 suppression reopens the window for effective rehabilitation after stroke in mice

**DOI:** 10.1126/sciadv.aed9140

**Published:** 2026-11-13

**Authors:** Toru Nakanishi, Takahide Itokazu, Hiroshi Tsujioka, Nao Shibuya, Hirofumi Morishita, Toshihide Yamashita

**Affiliations:** ^1^ Department of Neuro-Medical Science, Graduate School of Medicine, The University of Osaka, Suita, Osaka, Japan.; ^2^ Department of Molecular Neuroscience, Graduate School of Medicine, The University of Osaka, Suita, Osaka, Japan.; ^3^ Department of Psychiatry, Neuroscience, and Ophthalmology, Icahn School of Medicine at Mount Sinai, New York, NY, USA.; ^4^ Department of Mental Disorder Research, National Institute of Neuroscience, National Center of Neurology and Psychiatry, Kodaira, Tokyo, Japan.; ^5^ WPI Immunology Frontier Research Center, The University of Osaka, Osaka, Japan.; ^6^ Graduate School of Frontier Bioscience, The University of Osaka, Suita, Osaka, Japan.

## Abstract

Rehabilitation is an effective and reliable approach for promoting motor recovery after stroke. However, its efficacy decreases during the transition from the subacute to the chronic phase after stroke, posing a major barrier to complete functional recovery. This limited efficacy during the chronic phase is believed to result from declining neural plasticity. However, the molecular and cellular mechanisms regulating neural plasticity and its temporal changes after stroke remain unclear. To address this gap, we established a focal ischemic stroke mouse model that allows causal interrogation of the mechanisms responsible for the reduced efficacy of rehabilitation in the chronic phase. Using this model, we identified Lynx1, an endogenous negative allosteric modulator of nicotinic acetylcholine receptors, as a negative regulator of post-stroke neural plasticity. *Lynx1* expression in layer 5 pyramidal neurons was transiently down-regulated and returned to normal levels in the peri-infarct area. Longitudinal intravital imaging revealed that Lynx1 suppression promoted synaptic clustering with increased dendritic spine turnover in the peri-infarct area, an emerging structural basis for memory formation. As anticipated, Lynx1 suppression improved spontaneous motor recovery and the efficacy of rehabilitation in the chronic phase after stroke. Furthermore, we developed a *Lynx1*-targeting antisense oligonucleotide and found that its administration during the chronic phase after stroke enhanced the efficacy of rehabilitation. These findings suggest that dendritic spine dynamics in the peri-infarct area are a key determinant of rehabilitation responsiveness through the regulation of clustered spine formation and that Lynx1 may represent a promising therapeutic target for augmenting dendritic plasticity after stroke.

## INTRODUCTION

Stroke is a leading cause of long-term disability and death. Annually, approximately 12 million people experience stroke, and its global prevalence continues to increase (*1*). Ischemic stroke occurs owing to occlusion or severe stenosis of the cerebral artery and accounts for nearly 70% of all stroke cases (*2*). Although intravenous thrombolysis and thrombectomy are effective in the acute phase, these treatments are applicable to only 5 to 10% of patients (*3*). After the acute phase, rehabilitation is one of the most effective and widely used approaches for promoting motor recovery. However, the efficacy of rehabilitation decreases as stroke progresses from the subacute to the chronic phase (*4*), which is a major barrier to complete functional recovery in patients with stroke. The limited efficacy of rehabilitation in the chronic phase is considered to result from reduced neural plasticity, which may be coordinated by a balance between positive and negative factors (*5*). However, the molecular and cellular mechanisms that regulate neural plasticity and its temporal changes after stroke are not fully understood.

Dendritic spines are highly dynamic protrusions that emanate from dendrites and primarily receive excitatory synaptic inputs. As they can be newly formed and disappear in an activity-dependent manner, structural changes in dendritic spines have been implicated in various forms of learning and memory (*6*, *7*). Higher levels of baseline spine turnover are correlated with a greater capacity for subsequent learning (*8*, *9*). Moreover, selective light-activated shrinkage of learning-induced new or recently potentiated spines impairs learned motor behaviors, indicating a causal relationship between newly formed spines and new motor tasks (*10*). After stroke, dendritic spines are rearranged on the apical dendrites of layer 5 pyramidal neurons adjacent to the infarct area, a process that may contribute to sensorimotor recovery by reorganizing the remaining neural circuits (*5*, *11*). Notably, dendritic spine turnover is transiently enhanced in the subacute phase and gradually declines in the chronic phase after stroke (*12*, *13*), suggesting that dendritic spine dynamics undergo temporal changes in the peri-infarct area. Moreover, recent studies have indicated that rehabilitation facilitates motor recovery after stroke by modifying dendritic spine morphology of layer 5 pyramidal neurons in the peri-infarct area (*14*, *15*). Therefore, we hypothesized that the efficacy of rehabilitation depends on dendritic spine dynamics in the peri-infarct area, which may determine whether dendritic spines are receptive to activity-dependent structural modifications induced by rehabilitation.

Here, we aimed to determine whether the enhancement of spine turnover in the peri-infarct area facilitates rehabilitation-mediated motor recovery in the chronic phase and spontaneous motor recovery after stroke. We identified Lynx1, an endogenous negative allosteric modulator of nicotinic acetylcholine receptors (nAChRs), as a negative regulator of spine turnover of layer 5 pyramidal neurons in the peri-infarct area. Given the role of nAChR signaling in activity-dependent synaptic plasticity (*16*, *17*), we also examined whether nAChR signaling contributes to the effect of Lynx1 suppression on rehabilitation responsiveness. Furthermore, we demonstrated that Lynx1 suppression promoted clustered spine formation and improved the efficacy of rehabilitation in the chronic phase following stroke. Together with these observations, we propose that dendritic spine dynamics in the peri-infarct area constitute a key determinant of rehabilitation responsiveness through the regulation of clustered spine formation after stroke and that Lynx1 may serve as a promising therapeutic target for augmenting dendritic plasticity.

## RESULTS

### Efficacy of rehabilitation decreases with time after stroke

To validate the temporal changes in the efficacy of rehabilitation after stroke, we induced a focal cortical infarct in the sensorimotor cortex of mice using a photothrombotic (PT) stroke model, as indicated in Fig. 1A (right) (coordinates: first lesion, a rectangular cortical region spanning AP −1.0 to +1.0 mm and ML 1.7 to 3.7 mm relative to bregma; second lesion, a rectangular cortical region spanning AP −1.0 to +1.0 mm and ML 1.2 to 1.7 mm relative to bregma). Because injury to the sensorimotor cortex impairs dexterous forelimb control in mice (*18*), we used a single-pellet reaching task to evaluate rehabilitation-mediated forelimb motor recovery (Fig. 1, A and B). We also used the single-pellet reaching task for rehabilitation. Rehabilitative reaching training was initiated either 7 or 28 days after stroke and was performed in 10 sessions over 2 weeks. Behavioral analysis revealed a marked reduction in the success rate of the reaching task 7 days after stroke (Fig. 1, C and G). We found that rehabilitation that started 7 days after stroke significantly improved motor recovery (Fig. 1, C and D), whereas rehabilitation that started 28 days after stroke had no significant effect (Fig. 1, G and H), which is in line with previous findings (*19*). These results suggest a time-dependent decline in rehabilitation efficacy. Notably, a second injury to the medial area of the infarcted sensorimotor cortex eliminated the forelimb motor recovery achieved by rehabilitation in the subacute phase (Fig. 1E), suggesting a crucial role of the peri-infarct area in rehabilitation-mediated motor recovery after stroke.

**Fig. 1.**
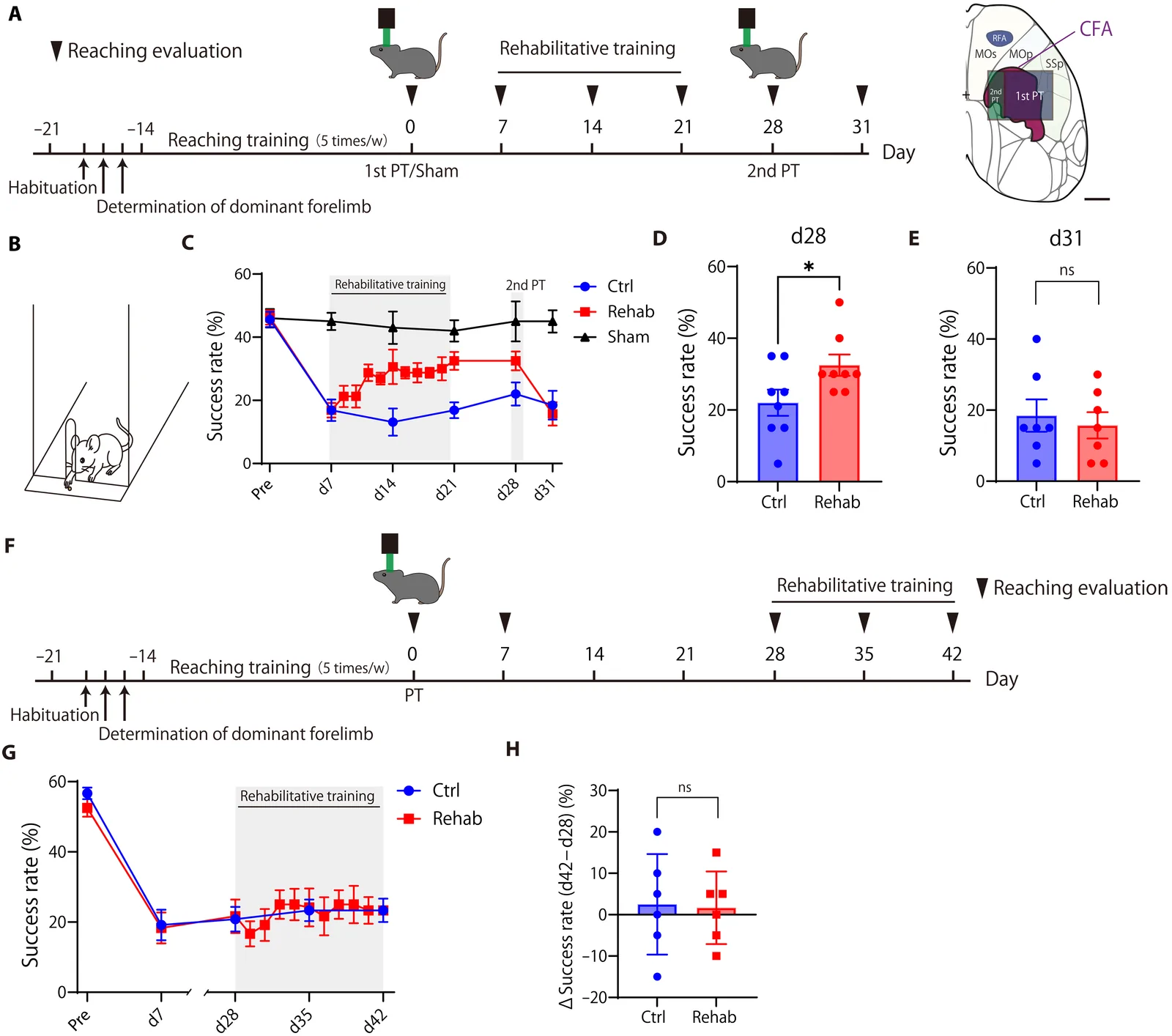
Rehabilitation efficacy decreases with time after stroke. (**A**) Experimental scheme. Motor function was evaluated once a week from day 7 to day 28, and on day 31, after stroke using the single-pellet reaching task. The single-pellet reaching training was also started as rehabilitation 7 days after stroke and was performed for 10 sessions over 2 weeks. Right: Schematic illustration of the intended PT lesions overlaid on a dorsal view of the Allen Mouse Brain Common Coordinate Framework (CCFv3) (*57*). The intended locations of the first and second PT lesions are shown in blue and green, respectively. The functionally defined caudal and rostral forelimb areas (CFA and RFA) are also indicated. The boundaries of the CFA and RFA were adapted from previous research (*58*). The plus sign (+) indicates bregma. Scale bar, 1 cm. (**B**) Schematic of the single-pellet reaching task. (**C**) Time course of the reaching success rate. Statistical comparisons were not performed because behavioral assessments were conducted at different time points across groups. (**D**) Reaching success rate on day 28 after stroke. Rehabilitation initiated 7 days after stroke significantly improved motor recovery (*n* = 8). Data are presented as means ± SEM. *P < 0.05; Student’s *t* test. (**E**) Reaching success rate on day 31 after stroke. The second PT lesion eliminated the forelimb motor recovery achieved through rehabilitation (*n* = 7). Data are presented as means ± SEM. ns, not significant, Student’s *t* test. (**F**) Experimental scheme. The rehabilitative reaching training started 28 days after stroke. (**G**) Time course of the reaching success rate. (**H**) Difference in the reaching success rate between day 28 and day 42. Rehabilitation initiated 28 days after stroke did not improve motor recovery (*n* = 6). Data are presented as means ± SEM (Student’s *t* test).

### *Lynx1* expression transiently decreases and then recovers in the peri-infarct area after stroke

To elucidate the molecular mechanisms regulating the temporal changes in the efficacy of rehabilitation after stroke, we conducted bulk RNA sequencing using RNA extracted from the infarcted sensorimotor cortex, including the surrounding peri-infarct area (Fig. 2a). To identify molecules that negatively regulate neural plasticity, we focused on genes that were down-regulated 7 days after stroke (d7) compared with sham and up-regulated 28 days after stroke (d28) compared with d7 (Fig. 2B). Gene set enrichment analysis revealed that the pathways associated with nicotine addiction showed significant changes in both comparisons (Fig. 2C). Thus, we investigated changes in the expression of nAChRs and their accessory proteins. We found that the expression of Lynx1, a member of the Ly6/uPAR/neurotoxin superfamily (*20*), was significantly altered after stroke (Fig. 2D). Lynx1 directly binds to nAChRs on the plasma membrane and functions as an endogenous negative allosteric modulator of these receptors (*21*). Moreover, *Lynx1* knockout mice exhibit enhanced synaptic plasticity and increased spine turnover in the adult visual cortex (*22*, *23*), suggesting that Lynx1 is associated with the structural plasticity of dendritic spines. On the basis of these previous findings, Lynx1 was prioritized for further investigation. Reverse transcription quantitative polymerase chain reaction (RT-qPCR) showed that *Lynx1* mRNA expression was significantly decreased in the sensorimotor cortex at 3, 7, and 14 days after stroke and returned to baseline levels at 21 days after stroke (Fig. 2E). Notably, this temporal change in *Lynx1* expression was also observed in yellow fluorescent protein (YFP)–positive layer 5 neurons in the peri-infarct area by fluorescence in situ hybridization in Thy1-YFP-H mice (Fig. 2, F and G). These findings prompted us to assume that Lynx1 is a candidate molecule that negatively regulates spine turnover in the peri-infarct area and the efficacy of rehabilitation after stroke.

**Fig. 2.**
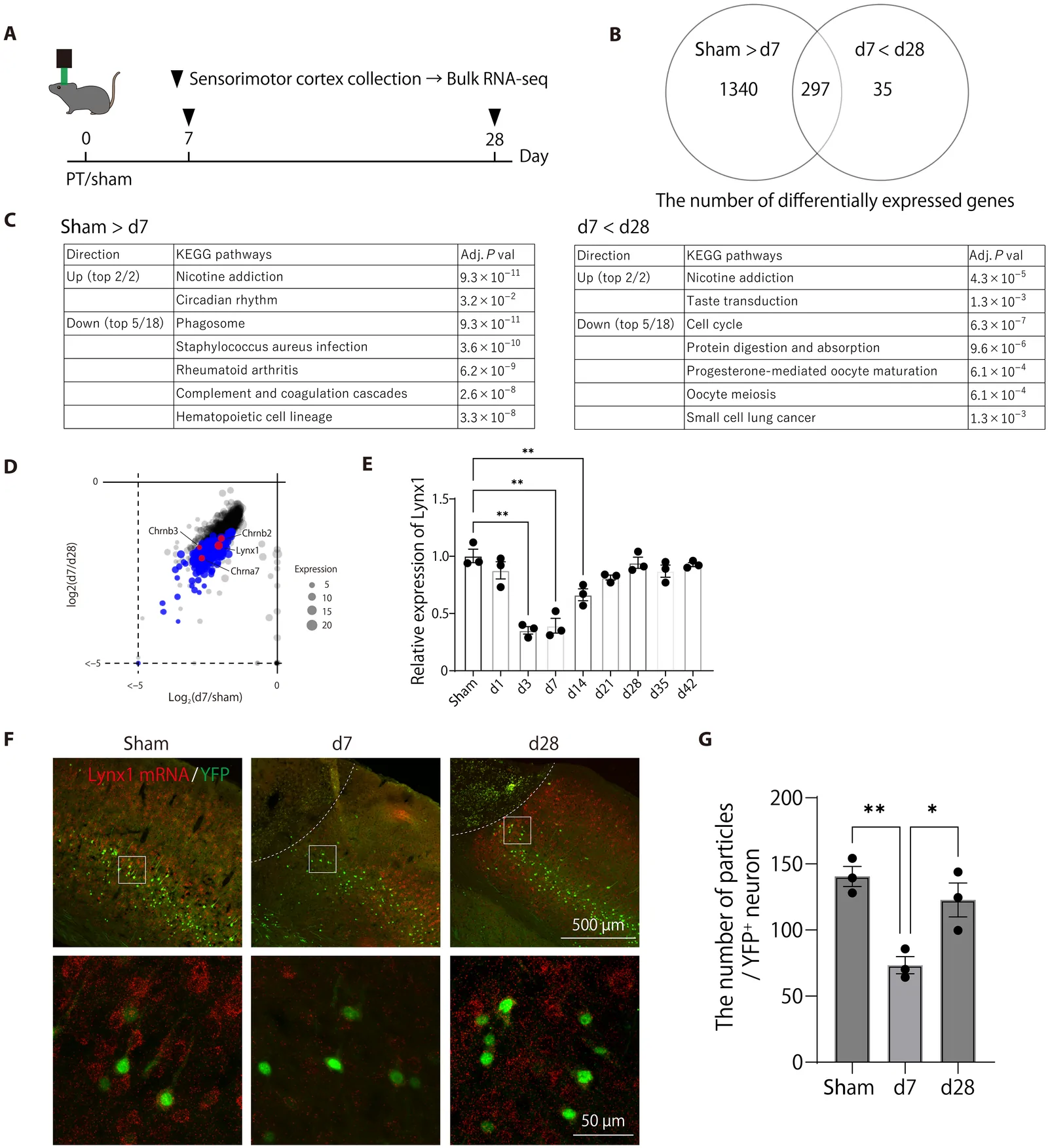
*Lynx1* expression transiently decreases and then recovers in the peri-infarct area after stroke. (**A**) Experimental time course of sampling. (**B**) Venn diagram showing the number of genes significantly down-regulated on day 7 after stroke (d7) compared with sham and significantly up-regulated on day 28 after stroke (d28) compared with d7. The overlap indicates the 297 genes meeting both criteria. (**C**) Significantly enriched Kyoto Encyclopedia of Genes and Genomes (KEGG) pathways on d7 compared with sham and on d28 compared with d7. (**D**) Genes shown in the Venn diagram (B) are plotted. The horizontal and vertical axes represent the log_2_ fold changes on d7 compared with sham and on d28 compared with d7, respectively. Blue dots indicate genes in the overlapping area of the two circles in the Venn diagram. Red dots indicate genes related to nicotinic acetylcholine signaling. Dot size indicates the expression level. Genes with fold changes of ≤−5 were truncated at −5. (**E**) *Lynx1* mRNA expression in the sensorimotor cortex 1, 3, 7, 14, 21, 28, 35, and 42 days after stroke or sham treatment (*n* = 3). Data are presented as means ± SEM. ^**^P < 0.01, one-way analysis of variance (ANOVA) followed by the Tukey-Kramer test. (**F**) Representative images showing *Lynx1* mRNA signals (red) in YFP-labeled pyramidal neurons in the sensorimotor cortex of sham and post-stroke mice. Lower panels show higher-magnification images of the boxed region in the top panels. Dashed lines outline the infarcted areas. Scale bars, 500 μm (top panels) and 50 μm (bottom panels). (**G**) Number of particles per YFP-positive neuron within 500 μm of the infarct border (*n* = 3). Data are presented as means ± SEM. **P < 0.01 and *P < 0.05, one-way ANOVA followed by the Tukey-Kramer test.

### *Lynx1* KD promotes spine turnover in the peri-infarct area after stroke

To investigate whether Lynx1 regulates spine turnover after stroke, we sought to suppress *Lynx1* expression in the peri-infarct cortex. A previous study showed that constitutive *Lynx1* knockout mice exhibited abnormal maturation of prefrontal attentional circuits and impaired attentional behavior (*24*). Furthermore, our results demonstrated that *Lynx1* expression was transiently down-regulated in the peri-infarct area after stroke (Fig. 2, F and G). Therefore, we adopted an adeno-associated virus (AAV)–mediated knockdown (KD) strategy to avoid potential developmental effects associated with constitutive knockout while restricting Lynx1 suppression to the adult peri-infarct cortex. In addition, because transient down-regulation of *Lynx1* was observed in YFP-positive pyramidal neurons in Thy1-YFP-H mice after stroke (Fig. 2, F and G), we constructed AAV vectors expressing *Lynx1* short hairpin RNA (shRNA) or control shRNA together with turbo red fluorescent protein (tRFP) under the CamkIIα promoter to selectively target excitatory neurons (Fig. 3A). We then validated the KD efficiency in vivo by RT-qPCR. *Lynx1* mRNA expression was significantly suppressed by *Lynx1* shRNA at 7 and 28 days after AAV injection (Fig. 3, B and C) and at 28 days after stroke (35 days after AAV injection) (Fig. 3D). We also confirmed that *Lynx1* KD did not affect infarct volume after stroke (Fig. 3, E and F). AAV-mediated tRFP expression under the CamkIIα promoter was predominantly observed in excitatory neurons rather than inhibitory neurons in the peri-infarct area after stroke (Fig. 3, G and H), consistent with the result of a previous study (*25*).

**Fig. 3.**
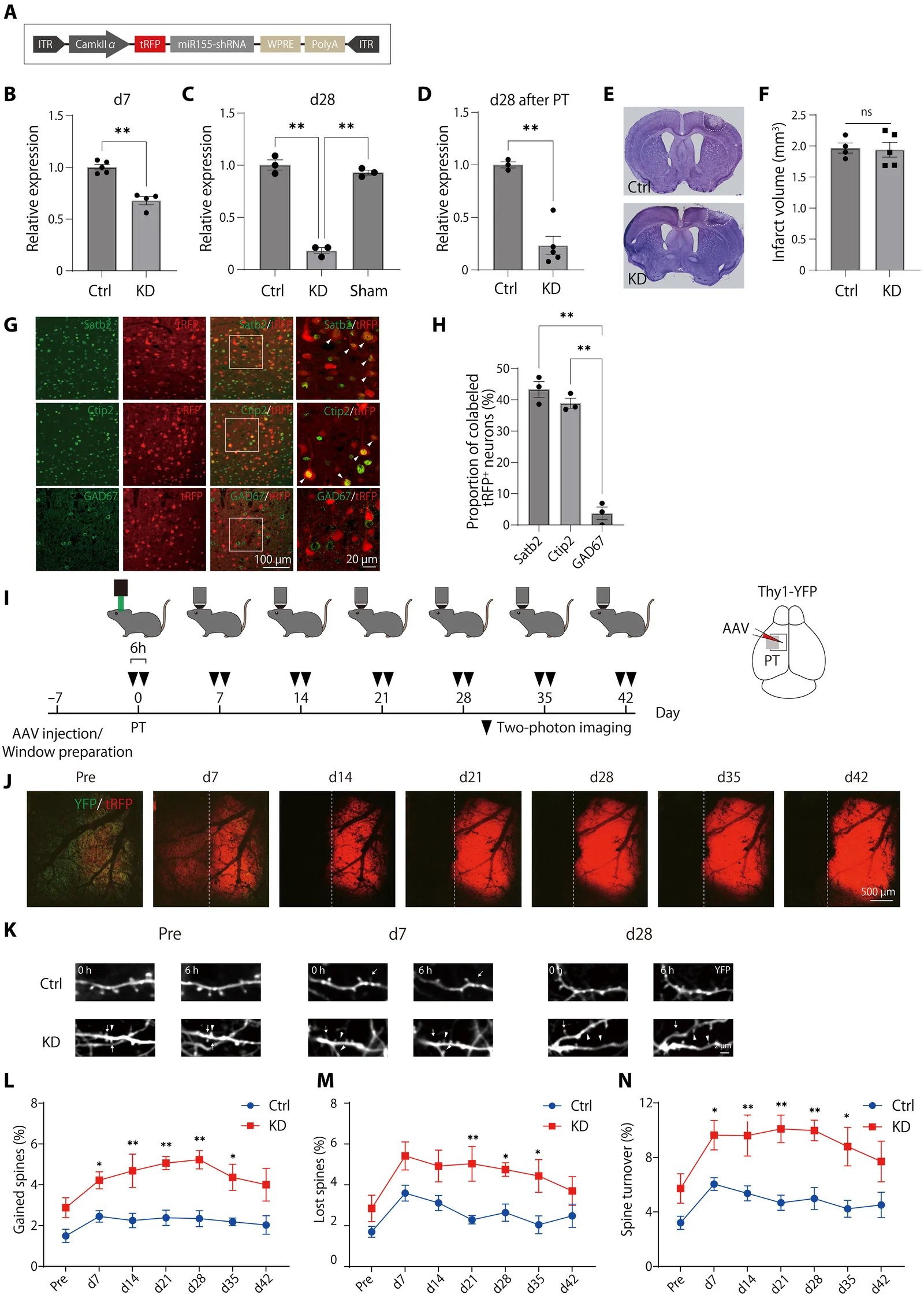
*Lynx1* KD promotes spine turnover in the peri-infarct area after stroke. (**A**) Schematic of AAV vectors. (**B** to **D**) *Lynx1* mRNA expression in the sensorimotor cortex 7 days (B) and 28 days (C) after AAV injection, and 28 days after stroke (D) (*n* = 3 to 5). Data are presented as means ± SEM. **P < 0.01 and *P < 0.05; Student’s *t* test (B and D) and one-way ANOVA followed by the Tukey-Kramer test (C). (**E**) Representative brain images from control and *Lynx1* KD mice after stroke. Dashed lines outline infarcted areas. (**F**) Infarct volume in control and *Lynx1* KD mice (*n* = 4 to 5). Data are presented as means ± SEM (Student’s *t* test). (**G**) Representative images of Satb2, Ctip2, and GAD67 immunostaining in AAV-injected mice. Rightmost panels show higher-magnification images of boxed regions. Arrowheads indicate representative tRFP-positive cells co-labeled with each marker. Scale bars, 100 μm (low magnification) and 20 μm (high magnification). (**H**) Proportion of tRFP-positive neurons co-labeled with each marker (*n* = 3). Data are presented as means ± SEM. **P < 0.01; one-way ANOVA followed by the Tukey-Kramer test. (**I**) Experimental scheme for longitudinal intravital imaging. Imaging was conducted weekly for up to 42 days after stroke in Thy1-YFP mice, with two sessions 6 hours apart at each time point. (**J**) Longitudinal images of the peri-infarct area. Scale bar, 500 μm. (**K**) Representative longitudinal images of YFP-positive dendritic spines acquired 6 hours apart. Arrows indicate eliminated spines. Arrowheads indicate newly formed spines. Scale bar, 2 μm. (**L** to **N**) Ratios of gained spines (L), lost spines (M), and spine turnover (N) at each time point (Ctrl, *n* = 5 to 6; KD, *n* = 3 to 5). Data are presented as means ± SEM. **P < 0.01 and *P < 0.05; mixed-effects model followed by Šídák's multiple comparison test.

Using these AAV-shRNAs, we performed longitudinal intravital imaging of dendritic spines in the peri-infarct area once a week for up to 42 days after stroke in Thy1-YFP mice (Fig. 3, I and J). Seven days before stroke, we injected AAV-shRNA into the medial area of the sensorimotor cortex adjacent to the putative infarct region and prepared a cranial window because this preserved area contributed to rehabilitation-mediated functional recovery after stroke (Fig. 1E). As it was difficult to follow the same dendrites between the observation time points owing to edema and vascular remodeling during the acute and subacute phases, we quantified the morphological changes in the spines occurring over 6 hours of imaging sessions at each time point, in accordance with a previous study (*12*). The mean number of dendritic spines analyzed per mouse at each imaging session ranged from 299.0 to 458.5 in the Ctrl group and from 206.0 to 488.6 in the KD group. Most dendritic spines were stable over the 6-hour imaging period immediately before stroke (pre) (Fig. 3K). Spine turnover increased in both groups 7 days after stroke, followed by a gradual decrease in control mice (Fig. 3K), consistent with previous observations (*12*, *13*). Notably, spine gain, loss, and turnover rates were significantly higher in *Lynx1* KD mice than in control mice after stroke (Fig. 3, L to N). These results indicate that Lynx1 negatively regulates spine turnover after stroke.

### *Lynx1* KD promotes motor recovery after stroke

Next, we performed a behavioral analysis to investigate whether Lynx1 suppression facilitates motor recovery after stroke. After pretraining, we injected AAV-shRNA into the medial area of the sensorimotor cortex and induced ischemic stroke in mice (Fig. 4A). The success rate sharply decreased on d7, followed by partial improvement in both groups. Notably, *Lynx1* KD mice showed better motor performance than control mice after stroke (Fig. 4B). This suggests that the promotion of spine turnover in the peri-infarct area by Lynx1 suppression may have a beneficial role in spontaneous motor recovery.

**Fig. 4.**
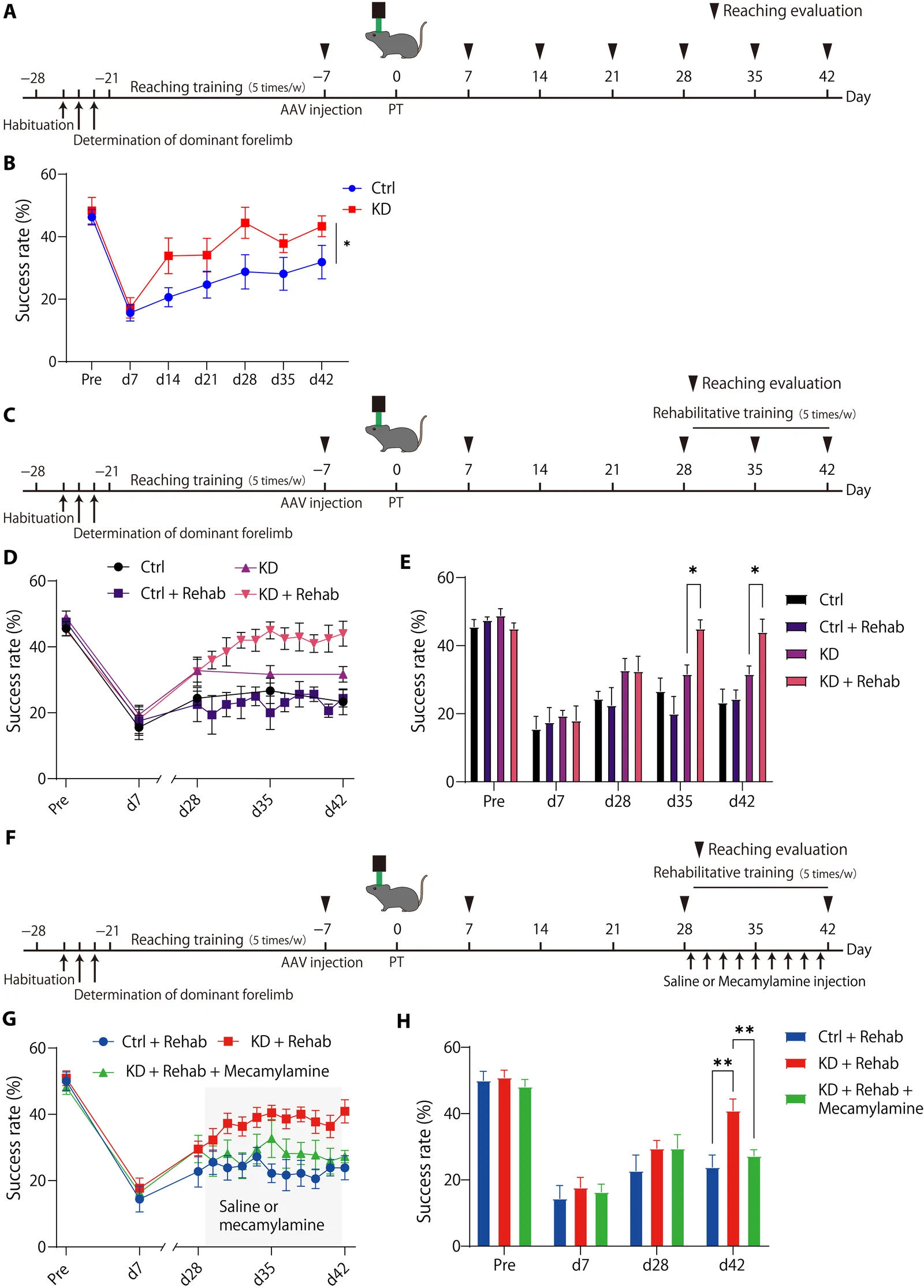
*Lynx1* KD promotes motor recovery after stroke. (**A**) Experimental time course. AAV expressing shRNA was injected into the sensorimotor cortex after pretraining. PT was induced 7 days after AAV injection. Motor function was assessed before AAV injection and weekly from d7 to d42 after stroke. (**B**) Time course of the reaching success rate (Ctrl, *n* = 8; KD, *n* = 9). Data are presented as means ± SEM. *P < 0.05. Asterisks indicate a significant main effect of group by two-way repeated measures ANOVA. (**C**) Experimental time course. Rehabilitative training started on d28 after stroke and was performed for 10 sessions over 2 weeks. (**D**) Time course of the reaching success rate. Statistical comparisons were not performed because assessment time points differed across groups. (**E**) Reaching success rate at common assessment time points (Ctrl, *n* = 9; Ctrl + Rehab, *n* = 8; KD, *n* = 9; KD + Rehab, *n* = 10). Data are presented as means ± SEM. *P < 0.05, two-way repeated measures ANOVA followed by the Bonferroni test. (**F**) Experimental time course. Rehabilitative training started on d28 after stroke and was performed for 10 sessions over 2 weeks. Saline or mecamylamine was injected intraperitoneally 30 min before each training session, except the final session. (**G**) Time course of the reaching success rate. Statistical comparisons were not performed because the data collected during rehabilitation were included to illustrate the time course of motor recovery. (**H**) Reaching success rate at behavioral assessment time points (Pre, 7, 28, and 42 days after stroke) (Ctrl + Rehab, *n* = 9; KD + Rehab, *n* = 11; KD + Rehab + Mecamylamine, *n* = 11). Data are presented as means ± SEM. **P < 0.01, two-way repeated measures ANOVA followed by the Bonferroni test.

The efficacy of rehabilitation after stroke decreases as stroke progresses from the subacute to the chronic phase in rodents and humans (*4*, *19*). Therefore, we assessed whether *Lynx1* KD enhances the efficacy of intensive motor rehabilitation for forelimb motor recovery in the chronic phase after stroke. Rehabilitative reaching training was initiated 28 days after stroke and was performed in 10 sessions over 2 weeks (Fig. 4C). Behavioral analysis revealed that rehabilitation did not have a significant effect on motor recovery in control mice, which is consistent with the result of a previous study (*19*), whereas rehabilitation in the chronic phase had a synergistic effect on forelimb motor recovery in *Lynx1* KD mice (Fig. 4, D and E). These results indicate that Lynx1 suppression enhances the efficacy of rehabilitation in the chronic phase after stroke. To examine whether nAChR signaling contributes to the beneficial effect of *Lynx1* KD, we administered mecamylamine, a nonselective nAChR antagonist, before each rehabilitative training session (Fig. 4F). Mecamylamine abolished the enhancement of rehabilitation-mediated motor recovery induced by *Lynx1* KD (Fig. 4, G and H). These findings suggest the involvement of nAChR signaling in the beneficial effect of *Lynx1* KD. To assess potential nonspecific behavioral effects of mecamylamine on task-related behavior, we additionally compared reach initiation latency, reach extension duration, and body weight between the KD + Rehab and KD + Rehab + Mecamylamine groups during the ninth rehabilitation session. No significant differences were observed between the two groups in reach initiation latency (KD + Rehab, 0.70 ± 0.08 s; KD + Rehab + Mecamylamine, 0.79 ± 0.09 s; *P* = 0.478), reach extension duration (KD + Rehab, 133 ± 5 ms; KD + Rehab + Mecamylamine, 142 ± 7 ms; *P* = 0.292), or body weight (KD + Rehab, 23.01 ± 0.28 g; KD + Rehab + Mecamylamine, 22.43 ± 0.42 g; *P* = 0.257) (means ± SEM; Student’s *t* tests).

### *Lynx1* KD promotes clustered spine formation during rehabilitation

To further investigate the mechanism underlying the improvement in the efficacy of rehabilitation in the chronic phase in *Lynx1* KD mice, we performed longitudinal intravital imaging of dendritic spines in the peri-infarct area between 28 and 42 days after stroke with or without Lynx1 suppression (Fig. 5A). As a previous study showed that a high spine turnover rate led to clustered spine formation and improved memory performance (*9*), we evaluated the rate of clustered spine formation and spine turnover rate. In *Lynx1* KD mice, spine turnover significantly increased between 28 and 35 days after stroke compared with control mice (Fig. 5, B to E). We then calculated the clustered spine ratio by dividing the number of newly formed spines located within 5 μm from the nearest new spine by the total number of newly formed spines (Fig. 5F). The clustered spine ratio at d35 was significantly higher in *Lynx1* KD mice (Fig. 5f). Notably, resampling analysis of data from control mice showed that clustering would occur randomly in 18.8%, which was close to the observed value of 17.9% (*P* > 0.05; Fig. 5G). In contrast, resampling analysis of data from *Lynx1* KD mice revealed that clustering of newly formed spines would occur by chance in 29.8%, whereas the observed proportion of clustered spines was 42.6% (*P* < 0.05; Fig. 5G). This analysis indicated that the enhanced clustering in *Lynx1* KD mice was not solely due to increased spine formation. Moreover, the clustered spine ratio was robustly correlated with the success rate of the reaching task on d42 (Fig. 5H). These results suggest that Lynx1 suppression promotes rehabilitation responsiveness by regulating clustered spine formation after stroke.

**Fig. 5.**
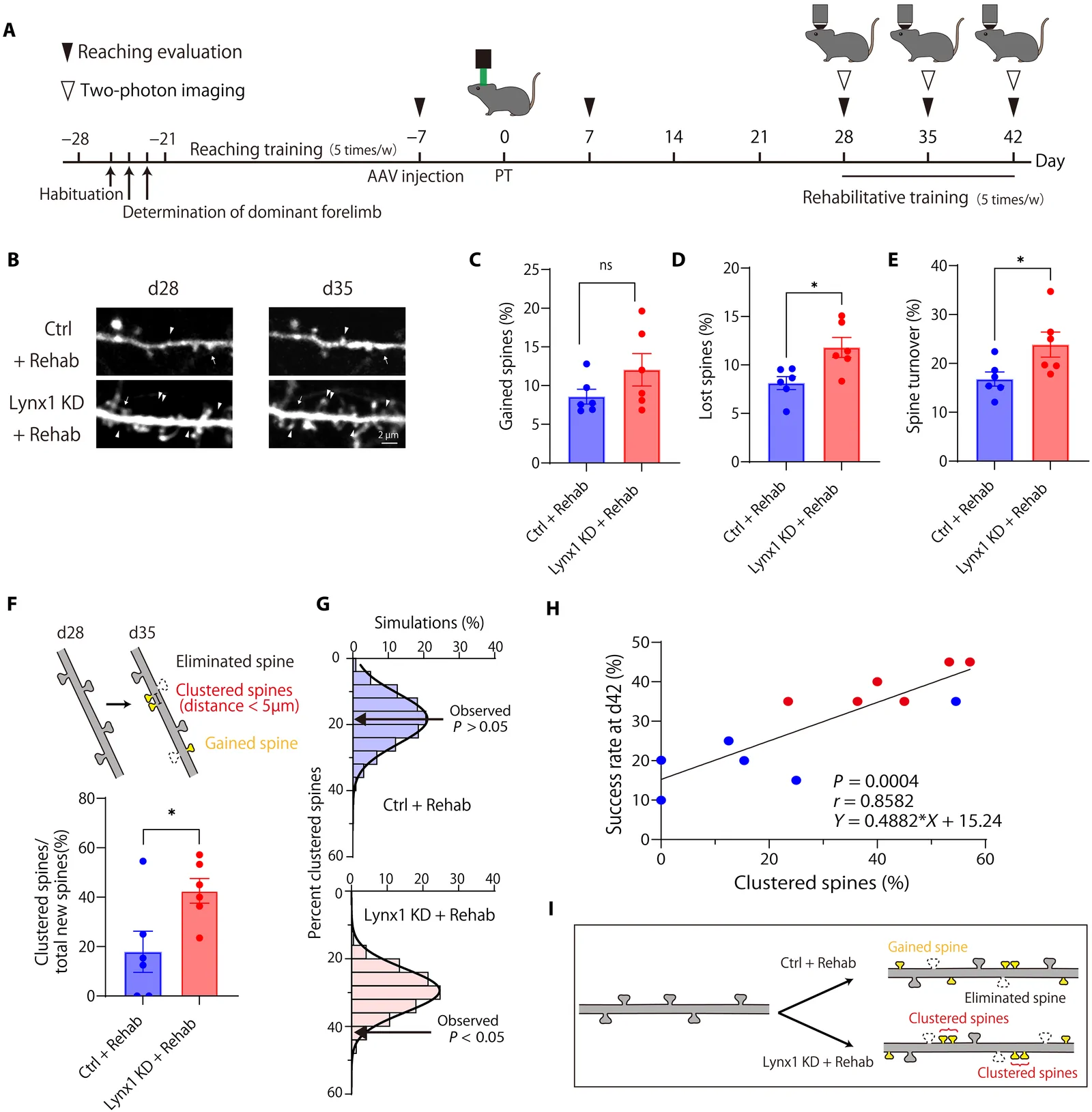
*Lynx1* KD promotes clustered spine formation during rehabilitation. (**A**) Experimental time course. AAV expressing shRNA was injected into the sensorimotor cortex after pretraining. PT was induced 7 days after AAV injection. Rehabilitative training started on d28 after stroke and was performed for 10 sessions over 2 weeks. Motor function was assessed before AAV injection and on days 7, 28, 35, and 42 after stroke. (**B**) Representative longitudinal images of YFP-positive dendritic spines on d28 and d35. Arrows indicate eliminated spines. Arrowheads indicate newly formed spines. Scale bar, 2 μm. (**C** to **E**) Ratios of gained spines (c), lost spines (d), and spine turnover (e) between d28 and d35 (*n* = 6). Data are presented as means ± SEM. *P < 0.05, Student’s *t* test. (**F**) Ratio of clustered spines between d28 and d35 (*n* = 6). Newly formed spines were classified as clustered when the nearest newly formed spine was within 5 μm. Data are presented as means ± SEM. *P < 0.05; Student’s *t* test. (**G**) Histogram showing the percentage distribution of clustered spine ratios from 10,000 simulations of randomized newly formed spine positions. The arrow indicates the observed clustered spine ratio. The black curve represents the Gaussian fit. In control mice, spine clustering was not higher than chance (mean of the Gaussian fit = 18.8%; observed = 17.9%; *n* = 6; *P* > 0.05). In *Lynx1* KD mice, spine clustering was greater than chance (mean of the Gaussian fit = 29.8%; observed = 42.6%; *n* = 6; *P* < 0.05). (**H**) Correlation between the clustered spine ratio and reaching success rate on d42 (*n* = 12; *P* < 0.05, Spearman’s correlation coefficient test). (**I**) Schematic illustration of dendritic spine dynamics in the control and *Lynx1* KD groups during rehabilitation.

### *Lynx1* KD by ASO facilitates the effect of rehabilitation after stroke

Last, we intended to provide evidence for therapeutics targeting *Lynx1* in stroke based on our results. Considering potential clinical application, the safety and efficacy of Lynx1 inhibition after stroke onset should be assessed. Therefore, we used an intracerebroventricular (ICV) injection of an antisense oligonucleotide (ASO) targeting *Lynx1*, flanked by 5 base pair of 2′-MOE–modified nucleotides, because of its safety and rapid mRNA degradation (Fig. 6A). To optimize the ASO sequence, five ASOs targeting *Lynx1* and a negative control ASO were directly injected into the motor cortex of mice. ASO #5 (hereafter referred to as ASO-Lynx1) showed the highest KD efficiency 7 days after injection (Fig. 6B). ASO-Lynx1 also induced significant *Lynx1* KD in the motor cortex 7 days after ICV injection (Fig. 6C).

**Fig. 6.**
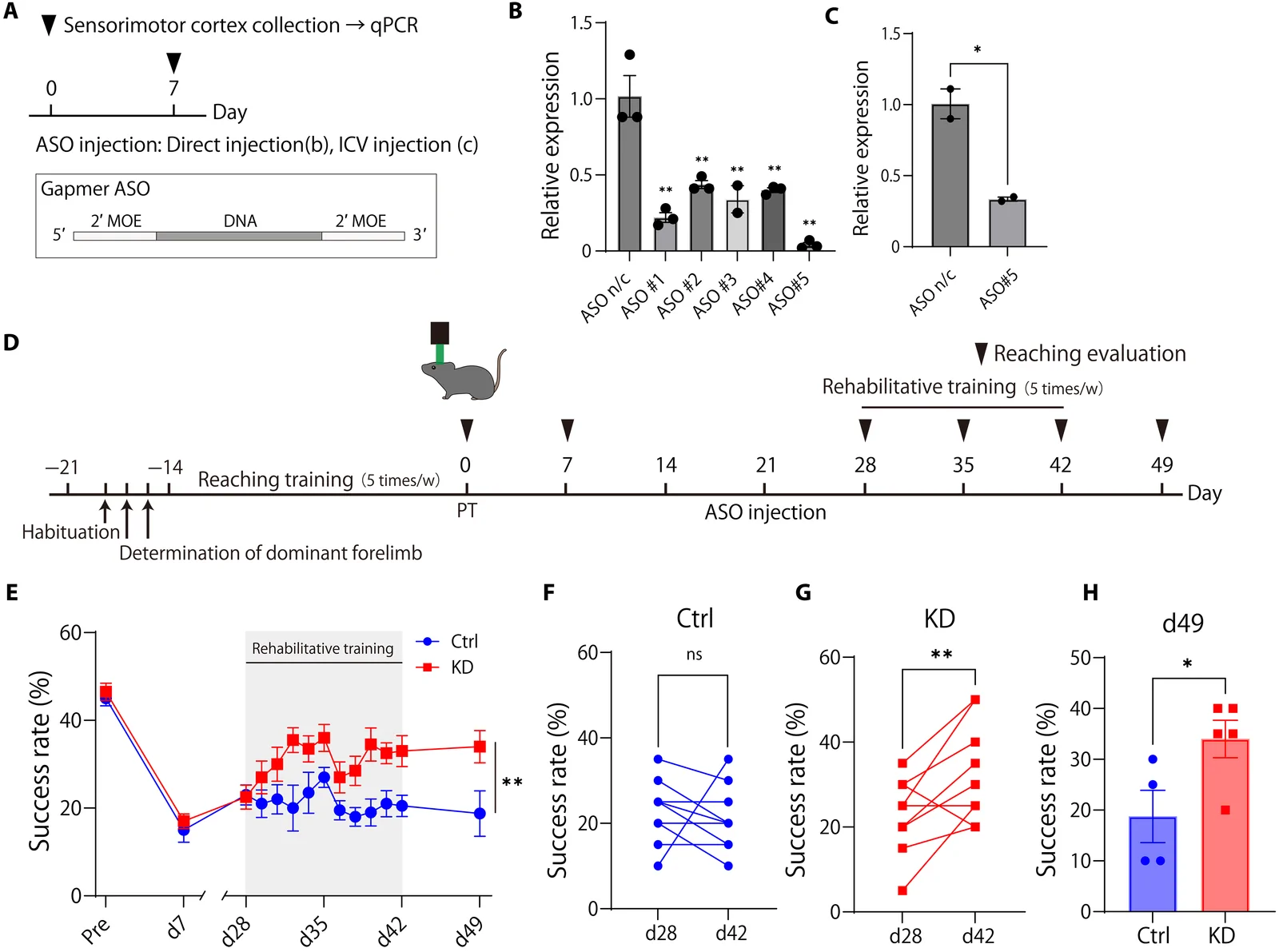
*Lynx1* KD by ASO enhances the efficacy of rehabilitation after stroke. (**A**) Experimental time course of sample collection. Seven days after gapmer ASO injection, the sensorimotor cortices were collected. (**B** and **C**) Quantification of *Lynx1* mRNA by real-time qPCR in the sensorimotor cortex 7 days after ASO administration by direct cortical (B) or ICV injection (C) (*n* = 2 to 3). Data are presented as means ± SEM. **P < 0.01 and *P < 0.05, one-way ANOVA followed by the Tukey-Kramer test (B) and Student’s *t* test (C). (**D**) Experimental time course. PT was induced after pretraining. ASO was administered by ICV injection 21 days after stroke. The single-pellet reaching training started as rehabilitation 28 days after stroke and was performed for 10 sessions over 2 weeks. Motor function was assessed before stroke and on d7, d28, d35, d42, and d49 after stroke using the single-pellet reaching task. (**E**) Time course of the reaching success rate before and after stroke. *Lynx1* KD mice showed better motor performance than control mice after rehabilitation (*n* = 10). Data are presented as means ± SEM. *P < 0.05. Asterisks indicate a significant main effect of group by two-way repeated measures ANOVA. (**F** and **G**) Reaching success rates between d28 and d42. Rehabilitation initiated 28 days after stroke improved motor recovery in *Lynx1* KD mice (*n* = 6). Data are presented as means ± SEM. *P < 0.05, Student’s *t* test. (**H**) Reaching success rate on d49. *Lynx1* KD mice showed better motor recovery (Ctrl, *n* = 4; KD, *n* = 5). Data are presented as means ± SEM. *P < 0.05, Student’s *t* test.

Using ASO-Lynx1 or a negative control ASO, we examined whether ICV administration of ASO-Lynx1 21 days after stroke enhances the efficacy of rehabilitation in the chronic phase using the single pellet reaching task (Fig. 6D). *Lynx1* KD mice showed better motor performance than control mice after stroke (Fig. 6E). We also compared the success rates between 28 and 42 days after stroke in *Lynx1* KD and control mice. Following rehabilitation, the success rate did not change in control mice, whereas it significantly increased in *Lynx1* KD mice (Fig. 6, F and G). Moreover, the improved motor function in *Lynx1* KD mice was maintained until 49 days after stroke, even 7 days after the end of rehabilitation (Fig. 6H). To determine whether *Lynx1* KD affects histological changes in the peri-infarct area, we performed immunohistochemical analyses using glial fibrillary acidic protein (GFAP), Iba1, NeuN, and the pan-axonal neurofilament marker SMI312 (fig. S1A). Quantification of the GFAP-positive area, Iba1-positive area, NeuN-positive cell density, and SMI312-positive axonal area revealed no significant differences between control and *Lynx1* KD mice (fig. S1, B to E). Together, these results suggest that ICV administration of ASO-Lynx1 after stroke onset enhances the efficacy of rehabilitation in the chronic phase.

## DISCUSSION

In the present study, we showed that enhanced dendritic spine dynamics in the peri-infarct area, induced by Lynx1 suppression, promoted the efficacy of rehabilitation by regulating clustered spine formation after stroke.

Several lines of evidence have shown that the structural plasticity of dendritic spines of layer 5 pyramidal neurons in the motor cortex plays an important role in the acquisition of new motor skills (*26*–*30*). Specifically, motor learning enhances spine formation, which is followed by increased spine elimination in the motor cortex. The rate of spine formation correlates with motor performance (*26*, *30*). Furthermore, the selective optogenetic elimination of learning-induced new spines impairs learned motor behaviors, indicating a causal relationship between newly formed spines and new motor tasks (*10*). Spine elimination is suggested to occur predominantly in spines exhibiting mistimed activity relative to postsynaptic output during motor learning and may contribute to enhancing the robustness of learned motor circuits (*31*). These studies suggest that morphological changes in the dendritic spines constitute the structural substrates for the acquisition of new motor skills. After stroke, structural changes in dendritic spines are also enhanced in the subacute phase, specifically in the peri-infarct area (*12*, *13*), where functional synaptic connections are reestablished to compensate for impaired neural circuits and restore damaged brain functions (*5*, *11*). A previous study showed that the formation of new synaptic connections in the peri-infarct area induced by optogenetic stimulation was associated with improved sensorimotor function after stroke (*32*). Therefore, promoted spine turnover in the peri-infarct area can contribute to the improvement of spontaneous motor recovery after stroke by facilitating reparative synaptic reconnections.

Here, we found that Lynx1 suppression enhanced spine turnover in the peri-infarct area, consistent with previous findings in the adult visual cortex of *Lynx1* knockout mice (*23*). Lynx1, a member of the Ly6/uPAR/neurotoxin superfamily (*20*), directly binds to nAChRs on the plasma membrane. It reduces the receptors’ sensitivity to acetylcholine, enhances desensitization, and delays recovery from the desensitized state (*33*, *34*), possibly by restricting the conformational transitions of nAChRs through interactions with the receptor C-loop, as suggested by molecular dynamics simulations (*35*). Moreover, Lynx1 regulates nAChR stoichiometry in the endoplasmic reticulum, thereby modulating the receptors’ functional properties (*36*). Lynx1 suppression relieves these regulatory effects, thereby increasing Ca^2+^ influx through nAChRs (*34*), which in turn activates multiple signaling pathways associated with both spine formation and spine shrinkage (*37*). Mice lacking the α7 or β2 nAChR subunits exhibit dendritic spine abnormalities, supporting the role of nAChR-mediated Ca^2+^ signaling in the structural regulation of dendritic spines (*38*, *39*). Moreover, nicotinic acetylcholine signaling induces either synaptic potentiation or depression, depending on the timing of cholinergic input relative to glutamatergic input (*16*, *17*). Because synaptic potentiation and depression are associated with the enlargement and shrinkage of dendritic spines, respectively (*40*, *41*)*,* and Lynx1 modulates nicotinic acetylcholine signaling while maintaining its temporal fidelity (*34*), Lynx1 suppression may promote spine turnover by amplifying the bidirectional effects of nicotinic acetylcholine signaling on synaptic plasticity.

Clustered spine formation during learning contributes to improved memory performance (*9*, *28*). These clustered spines are believed to facilitate the nonlinear integration of synaptic inputs and enhance neural plasticity (*42*). They may also provide the structural substrates for the formation of memory engrams (*42*). However, it remains unclear whether clustered spine formation contributes to motor recovery during post-stroke rehabilitation.

In this study, we found that rehabilitation in the chronic phase after stroke combined with Lynx1 suppression induced clustered spine formation and promoted rehabilitation-mediated motor recovery. Recent studies have demonstrated that acetylcholine is released not only in association with arousal and attention (*43*, *44*) but also in response to unexpected reward or punishment (*45*, *46*). These observations suggest that cholinergic modulation functions as a gating mechanism, allowing the spatiotemporally restricted enhancement of synaptic plasticity in response to salient inputs. The enhancement of motor learning by vagus nerve stimulation (VNS), which is mediated by acetylcholine signaling, is achieved only when VNS is delivered immediately after a successful trial, suggesting a role for acetylcholine as a reinforcement signal (*47*). Therefore, Lynx1 suppression likely enhances clustered spine formation by augmenting cholinergic gating while preserving its temporal fidelity in a context-dependent manner. This may contribute to the formation of more refined compensatory neural circuits in the peri-infarct area, resulting in improved motor function through rehabilitation after stroke. Further studies are required to confirm these hypotheses.

Exogenous enhancement of dendritic spine dynamics across brain areas can lead to the formation of aberrant neural circuits or the destabilization of neural circuits, which may impair motor recovery or the maintenance of acquired motor functions, respectively. However, we demonstrated that ICV administration of ASO-Lynx1 after stroke onset enhanced the efficacy of rehabilitation in the chronic phase, and the improved motor performance was maintained until 7 days after the end of rehabilitation (Fig. 6H). These results suggest that the promotion of dendritic spine motility by Lynx1 suppression is beneficial, without any noticeable side effects. In recent studies, several modifications that enable ASOs to cross the blood-brain barrier have been developed, including cholesterol-conjugated DNA/RNA heteroduplex oligonucleotides (*48*) and engineered transport vehicle-conjugated ASOs (*49*). Therefore, it would be interesting to assess the therapeutic effect of intravenous administration of ASO-Lynx1 with these modifications after stroke to increase the translational applicability of this therapeutic strategy in future studies.

In conclusion, we demonstrated that modulation of dendritic spine dynamics in the peri-infarct area reopens the time window for effective rehabilitation by regulating clustered spine formation after stroke. Moreover, Lynx1 may serve as a promising therapeutic target molecule for manipulating dendritic plasticity and reinstating the efficacy of rehabilitation in the chronic phase after stroke. These findings may lead to the development of new therapeutic approaches for chronic stroke.

## MATERIALS AND METHODS

### Animals

Adult C57BL/6 J male mice (age, 6 to 12 weeks; Japan SLC Inc.) and Thy1-YFP-H male mice (age, 6 to 12 weeks; the Jackson Laboratory, stock no. 003782, RRID: IMSR_JAX:003782) (*50*) were used in this study. Thy1-YFP-H mice were used for longitudinal in vivo two-photon imaging because sparse YFP expression in a subset of layer 5 pyramidal neurons enables repeated visualization of individual apical dendrites and dendritic spines (*12*, *13*, *50*). All experimental procedures were approved by the Institutional Ethics Committee of Osaka University (approval no. 04-042) and complied with the Osaka University Medical School Guidelines for the Care and Use of Laboratory Animals.

### PT stroke model

The mice were anesthetized by intraperitoneal injection of a mixture of butorphanol (0.5 mg/ml; Vetorphale, Meiji Seika Pharma), midazolam (0.4 mg/ml; Dormicum, Maruishi Pharma), and medetomidine (0.3 mg/ml; Domitor, Orion Pharma) dissolved in sterile water or with 1 to 2% (v/v) isoflurane (Pfizer). The skull was covered with aluminum foil, leaving the sensorimotor cortex exposed (2.0 mm by 0.5 mm, 2.0 mm by 2.0 mm, or 3.0 mm in diameter). After intravenous injection of 200 μl of rose bengal (330,000, Sigma-Aldrich; 10 mg/mlL) dissolved in saline, the exposed region was illuminated with a green collimated light-emitting diode light (M565L3, Thorlabs) for 15 min.

### Optical window preparation

For in vivo imaging, a cranial window was created using a previously reported open skull procedure with minor modifications (*51*). In brief, the mice were anesthetized with 1 to 2% (v/v) isoflurane (Pfizer) during the surgery. After the scalp was incised and removed, a custom-made headpost was attached to the skull overlying the sensorimotor cortex using dental acrylate (Sun Medical). After fixing the headpost to a custom-made clamp, a 2.5 mm–by–2.5 mm region on the parietal bone (AP, ±1.25 mm; ML, 0.5 to 3.0 mm from bregma) was marked. The marked region of the skull was carefully removed. The cranial window was then sealed with a #1 square coverslip (2.5 mm by 2.5 mm; Matsunami) and a #1 round coverslip (Φ3.8 mm; Matsunami) glued by cyanoacrylate (04613, Konishi).

### Two-photon imaging

For imaging sessions, the animals were anesthetized with 1 to 2% (v/v) isoflurane and fixed to a clamp. Two-photon imaging was performed using the Olympus FluoView FVMPE-RS multiphoton microscope system. A Spectra-Physics MaiTai HP DeepSee Ti:sapphire laser was tuned to 910 nm for YFP excitation, with the laser power at the back aperture set to ∼20 mW. All images were captured using either a 5× objective lens (MPLN5X) or a 25× water-immersion objective lens (XLPN25XWMP) equipped with an adapter (Thorlabs). Dendritic spine images were acquired up to a depth of 50 or 100 μm at a magnification of 5× zoom and taken in 1.0-μm *z*-steps. A pixel dwell time of 2 μs with a frame size of 1024 × 1024 pixels was used.

### Quantification of morphological changes in dendritic spines

During two-photon imaging, dendritic segments showing sufficient image quality for reliable spine identification and no gross morphological changes during the observation period were selected for quantitative analysis according to pre-established inclusion criteria (*12*, *13*). On these segments, protrusions extending ≥0.4 μm laterally from the dendritic shaft were counted as dendritic spines (*23*, *51*). If image quality no longer allowed reliable identification of dendritic spines, then subsequent imaging sessions from that animal were excluded from the analysis. The rates of spine gain, loss, and turnover were calculated by dividing the number of gained spines, lost spines, and spines that were either gained or lost between imaging sessions by the total number of spines present in the first session. A newly formed spine was classified as clustered when the nearest newly formed spine was within 5 μm (*9*). The number of clustered spines was divided by the total number of newly formed spines. The resampling analysis of clustering was performed in accordance with an earlier study with small modifications (*9*). Briefly, to assess whether clustered spine formation exceeded chance, we placed the observed number of newly formed spines at random positions along the measured segment length of the analyzed dendrites and computed the fraction of spines with a nearest-neighbor distance ≤5 μm. This randomization was repeated 10,000 times for each animal, and the values were averaged for each group.

### Single-pellet reaching task

The single-pellet reaching task was used to assess forelimb motor recovery after stroke (*18*) and as a task-specific rehabilitative intervention. The procedure followed that of a previous study, with minor modifications (*52*). Briefly, before stroke, the mice were trained to reach for pellets using the dominant forelimb 5 days per week for 2 weeks. Mice that achieved a success rate of at least 40% in the reaching task at the final pretraining session were included in subsequent experiments according to a pre-established inclusion criterion. A trial was scored as successful when the mouse reached for, grasped, and retrieved the pellet to its mouth without dropping it. The success rate was calculated as the number of successful reaches out of 20 trials. For rehabilitative training, mice performed the same single-pellet reaching task once daily, 5 days per week for 2 weeks (10 sessions in total). Each rehabilitation session consisted of 20 reaching trials and ended upon completion of the 20th trial. Video recordings (29.97 frames/s) from the ninth rehabilitation session were analyzed for reach initiation latency and reach extension duration. Reach initiation latency was defined as the time from pellet placement to the onset of the reaching movement and reach extension duration as the time from reach onset to maximal forelimb extension. For each mouse, reach initiation latency and reach extension duration were averaged across analyzed reaching trials.

### Drug administration

Mice were injected intraperitoneally with mecamylamine (5 mg/kg; 2843, Tocris), a noncompetitive, nonselective nAChR antagonist, or vehicle (saline) 30 min before each rehabilitative training session, as described previously (*53*).

### AAV purification and injection

pAAV-CamkIIα-tRFP-Lynx1 shRNA and pAAV-CamkIIα-tRFP-control shRNA were custom-built by VectorBuilder. The shRNA sequences were as follows: *Lynx1* (5′-AGCAAAGCCAGCACCGTTGCA-3′) and control (5′-AAATGTACTGCGCGTGGAGAC-3′). AAV1-CamkIIα-tRFP-shRNAs were purified using the AAVpro Purification Kit (Takara Bio Inc., Japan) according to the manufacturer’s protocol. Viral titers were measured using the AAVpro Titration Kit (Takara Bio Inc., Japan) with a QuantStudio 7 real-time PCR system (Applied Biosystems) and adjusted to 2.6 × 10^12^ gc/ml. A total of 0.4 μl per site of AAV1-CamkIIα-tRFP-shRNA was infused into the sensorimotor cortex (coordinates relative to bregma: +0.6 mm AP/1.2 mm ML, ±0 mm AP/1.2 mm ML, and − 0.6 mm AP/1.2 mm ML, each at a depth of 0.6 mm).

### Immunohistochemistry

The animals were deeply anesthetized and transcardially perfused with 4% paraformaldehyde (PFA) in 0.1 M phosphate buffer. Following perfusion, the brains were postfixed in 4% PFA at 4°C overnight and subsequently transferred to 30% sucrose in phosphate-buffered saline (PBS). The tissues were embedded in Tissue-Tek O.C.T. Compound (Sakura Finetek). The brains were cut into 20- or 30-μm-thick coronal sections using a cryostat and mounted on MAS-coated glass slides (Matsunami). For immunohistochemistry, the sections were washed three times with PBS and blocked with 5% bovine serum albumin/0.1% Triton X-100/PBS for 1 hour. Subsequently, the sections were incubated with primary antibodies overnight at 4°C. The primary antibodies used were as follows: guinea pig anti-SATB2 (327004, Synaptic Systems), rat anti-Ctip2 (ab18465, Abcam), mouse anti-GAD67 (MAB5406, Millipore), chicken anti-GFAP (ab4674, Abcam), rabbit anti-Iba1 (019-19741, Wako), mouse anti-NeuN (MAB377, Merck), and mouse anti-pan-axonal neurofilament marker (SMI312) (837904, BioLegend). The sections were subsequently washed three times with PBS, followed by incubation with secondary antibodies for 1 hour at room temperature in the dark. Alexa Fluor 488–conjugated donkey antibodies against guinea pig, rat, or mouse IgG; Alexa Fluor 488–conjugated goat antibodies against chicken; Alexa Fluor 568–conjugated goat antibodies against rabbit; and Alexa Fluor 647–conjugated goat antibodies against mouse (Invitrogen) were used as the secondary antibodies. All images were acquired using a confocal laser-scanning microscope (Olympus FV3000, Tokyo, Japan). The proportion of colabeled tRFP-positive neurons was calculated by dividing the number of tRFP-positive neurons colabeled with each cell marker by the total number of tRFP-positive neurons.

### Fluorescence in situ hybridization

Brain sections were prepared from Thy1-YFP-H mice, as described above. RNAscope probes targeting *Lynx1* [Advanced Cell Diagnostics (ACD), 449071] were used, and fluorescence in situ hybridization was performed using the RNAscope Multiplex Fluorescent Kit v1 (ACD, 320850) according to the manufacturer’s protocol.

### Quantitative polymerase chain reaction

The sensorimotor cortices of mice were dissected and homogenized in TRIzol reagent (Thermo Fisher Scientific). Total RNA was isolated and purified using the RNeasy Mini Kit (QIAGEN). The cDNA was synthesized using a High-Capacity cDNA Reverse Transcription Kit (Applied Biosystems). Primer sequences for *Lynx1* and *GAPDH* were those described previously (*54*). RT-qPCR was conducted using SYBR Green Master Mix (Thermo Fisher Scientific). *Lynx1* expression was normalized to *GAPDH*. PCR reactions and data analysis were performed using a QuantStudio 7 Flex Real-Time PCR System (Applied Biosystems).

### RNA sequencing

Total RNA was extracted from the sensorimotor cortex, as described above. Full-length cDNA was generated using a SMART-Seq HT Kit (Takara Bio, Mountain View, CA, USA) according to the manufacturer’s protocol. An Illumina library was prepared using the Nextera DNA Library Preparation Kit (Illumina, San Diego, CA, USA) according to SMARTer kit instructions. Sequencing was performed on an Illumina HiSeq 2500 sequencer (Illumina) in 75-base single-end mode. The transcript-level scaled TPM was quantified using the RNA pipeline ikra version 2.0.1 (*55*). Differential gene expression analysis and pathway enrichment were conducted using the integrative platform iDEP 2.0 (*56*).

### ASO injection

MOE gapmer ASOs targeting *Lynx1* were custom-built by Integrated DNA Technologies. The ASO sequences were as follows: #1, 5′-]TAGCCATCGTACACGGTTTC-3′; #2, 5′-TTGGTTGAGGACGCCATCTA-3′; #3, 5′-ACGTGCTTGCCGCAGAAGAT-3′; #4, 5′-TACACACTTCGTGCGGTGTT-3′; #5, 5′-GCCTATCTGGTCGGTGTCTA-3′; and control, 5′-CCTATAGGACTATCCAGGAA-3′. For direct administration into the sensorimotor cortex, 2 μl of ASO at 25 μg/μl (50 μg total) was injected. For ICV administration, 4 μl of ASO at 50 μg/μl (200 μg total) was injected into the lateral ventricle.

### Statistical analyses

All data are presented as means ± SEM. Statistical analyses were performed using GraphPad Prism version 9 (GraphPad Software). Statistical tests were selected according to the experimental design, the number of groups being compared, and whether repeated measurements were obtained from the same animals. Comparisons were performed using Student’s *t* test, Pearson’s correlation coefficient test, one-way analysis of variance (ANOVA) followed by Tukey-Kramer tests, two-way repeated measures ANOVA followed by Bonferroni tests, and mixed-effects model followed by Šídák's multiple comparison test. Outcome assessment and data analysis were performed by researchers blinded to the experimental conditions. Mice meeting the inclusion criteria were randomly assigned to the experimental groups. All experiments were conducted using independent biological replicates. The sample size (*n*) for each experiment is indicated in the corresponding figure legend. Sample sizes were determined on the basis of previous studies and our prior experience with similar experimental paradigms. A *P* value of < 0.05 was considered statistically significant.

## References

[1] V. L. Feigin, B. A. Stark, C. O. Johnson, G. A. Roth, C. Bisignano, G. G. Abady, M. Abbasifard, M. Abbasi-Kangevari, F. Abd-Allah, V. Abedi, A. Abualhasan, N. M. E. Abu-Rmeileh, A. I. Abushouk, O. M. Adebayo, G. Agarwal, P. Agasthi, B. O. Ahinkorah, S. Ahmad, S. Ahmadi, Y. Ahmed Salih, B. Aji, S. Akbarpour, R. O. Akinyemi, H. al Hamad, F. Alahdab, S. M. Alif, V. Alipour, S. M. Aljunid, S. Almustanyir, R. M. al-Raddadi, R. al-Shahi Salman, N. Alvis-Guzman, R. Ancuceanu, D. Anderlini, J. A. Anderson, A. Ansar, I. C. Antonazzo, J. Arabloo, J. Ärnlöv, K. D. Artanti, Z. Aryan, S. Asgari, T. Ashraf, M. Athar, A. Atreya, M. Ausloos, A. A. Baig, O. C. Baltatu, M. Banach, M. A. Barboza, S. L. Barker-Collo, T. W. Bärnighausen, M. T. U. Barone, S. Basu, G. Bazmandegan, E. Beghi, M. Beheshti, Y. Béjot, A. W. Bell, D. A. Bennett, I. M. Bensenor, W. M. Bezabhe, Y. M. Bezabih, A. S. Bhagavathula, P. Bhardwaj, K. Bhattacharyya, A. Bijani, B. Bikbov, M. M. Birhanu, A. Boloor, A. Bonny, M. Brauer, H. Brenner, D. Bryazka, Z. A. Butt, F. L. Caetano dos Santos, I. R. Campos-Nonato, C. Cantu-Brito, J. J. Carrero, C. A. Castañeda-Orjuela, A. L. Catapano, P. A. Chakraborty, J. Charan, S. G. Choudhari, E. K. Chowdhury, D. T. Chu, S. C. Chung, D. Colozza, V. M. Costa, S. Costanzo, M. H. Criqui, O. Dadras, B. Dagnew, X. Dai, K. Dalal, A. A. M. Damasceno, E. D'Amico, L. Dandona, R. Dandona, J. Darega Gela, K. Davletov, V. de la Cruz-Góngora, R. Desai, D. Dhamnetiya, S. D. Dharmaratne, M. L. Dhimal, M. Dhimal, D. Diaz, M. Dichgans, K. Dokova, R. Doshi, A. Douiri, B. B. Duncan, S. Eftekharzadeh, M. Ekholuenetale, N. el Nahas, I. Y. Elgendy, M. Elhadi, S. I. el-Jaafary, M. Endres, A. Y. Endries, D. A. Erku, E. J. A. Faraon, U. Farooque, F. Farzadfar, A. H. Feroze, I. Filip, F. Fischer, D. Flood, M. M. Gad, S. Gaidhane, R. Ghanei Gheshlagh, A. Ghashghaee, N. Ghith, G. Ghozali, S. Ghozy, A. Gialluisi, S. Giampaoli, S. A. Gilani, P. S. Gill, E. V. Gnedovskaya, M. Golechha, A. C. Goulart, Y. Guo, R. Gupta, V. B. Gupta, V. K. Gupta, P. Gyanwali, N. Hafezi-Nejad, S. Hamidi, A. Hanif, G. J. Hankey, A. Hargono, A. Hashi, T. S. Hassan, H. Y. Hassen, R. J. Havmoeller, S. I. Hay, K. Hayat, M. I. Hegazy, C. Herteliu, R. Holla, S. Hostiuc, M. Househ, J. Huang, A. Humayun, B. F. Hwang, L. Iacoviello, I. Iavicoli, S. E. Ibitoye, O. S. Ilesanmi, I. M. Ilic, M. D. Ilic, U. Iqbal, S. S. N. Irvani, S. M. S. Islam, N. E. Ismail, H. Iso, G. Isola, M. Iwagami, L. Jacob, V. Jain, S. I. Jang, S. K. Jayapal, S. Jayaram, R. Jayawardena, P. Jeemon, R. P. Jha, W. D. Johnson, J. B. Jonas, N. Joseph, J. J. Jozwiak, M. Jürisson, R. Kalani, R. Kalhor, Y. Kalkonde, A. Kamath, Z. Kamiab, T. Kanchan, H. Kandel, A. Karch, P. D. M. C. Katoto, G. A. Kayode, P. Keshavarz, Y. S. Khader, E. A. Khan, I. A. Khan, M. Khan, M. A. B. Khan, M. N. Khatib, J. Khubchandani, G. R. Kim, M. S. Kim, Y. J. Kim, A. Kisa, S. Kisa, M. Kivimäki, D. Kolte, A. Koolivand, S. L. Koulmane Laxminarayana, A. Koyanagi, K. Krishan, V. Krishnamoorthy, R. V. Krishnamurthi, G. A. Kumar, D. Kusuma, C. la Vecchia, B. Lacey, H. M. Lak, T. Lallukka, S. Lasrado, P. M. Lavados, M. Leonardi, B. Li, S. Li, H. Lin, R. T. Lin, X. Liu, W. D. Lo, S. Lorkowski, G. Lucchetti, R. Lutzky Saute, H. Magdy Abd el Razek, F. G. Magnani, P. B. Mahajan, A. Majeed, A. Makki, R. Malekzadeh, A. A. Malik, N. Manafi, M. A. Mansournia, L. G. Mantovani, S. Martini, G. Mazzaglia, M. M. Mehndiratta, R. G. Menezes, A. Meretoja, A. G. Mersha, J. Miao Jonasson, B. Miazgowski, T. Miazgowski, I. M. Michalek, E. M. Mirrakhimov, Y. Mohammad, A. Mohammadian-Hafshejani, S. Mohammed, A. H. Mokdad, Y. Mokhayeri, M. Molokhia, M. A. Moni, A. A. Montasir, R. Moradzadeh, L. Morawska, J. Morze, W. Muruet, K. I. Musa, A. J. Nagarajan, M. Naghavi, S. Narasimha Swamy, B. R. Nascimento, R. I. Negoi, S. Neupane Kandel, T. H. Nguyen, B. Norrving, J. J. Noubiap, V. E. Nwatah, B. Oancea, O. O. Odukoya, A. T. Olagunju, H. Orru, M. O. Owolabi, J. R. Padubidri, A. Pana, T. Parekh, E. C. Park, F. Pashazadeh Kan, M. Pathak, M. F. P. Peres, A. Perianayagam, T. M. Pham, M. A. Piradov, V. Podder, S. Polinder, M. J. Postma, A. Pourshams, A. Radfar, A. Rafiei, A. Raggi, F. Rahim, V. Rahimi-Movaghar, M. Rahman, M. A. Rahman, A. M. Rahmani, N. Rajai, P. Ranasinghe, C. R. Rao, S. J. Rao, P. Rathi, D. L. Rawaf, S. Rawaf, M. B. Reitsma, V. Renjith, A. M. N. Renzaho, A. Rezapour, J. A. B. Rodriguez, L. Roever, M. Romoli, A. Rynkiewicz, S. Sacco, M. Sadeghi, S. Saeedi Moghaddam, A. Sahebkar, K. M. Saif-Ur-Rahman, R. Salah, M. Samaei, A. M. Samy, I. S. Santos, M. M. Santric-Milicevic, N. Sarrafzadegan, B. Sathian, D. Sattin, S. Schiavolin, M. P. Schlaich, M. I. Schmidt, A. E. Schutte, S. G. Sepanlou, A. Seylani, F. Sha, S. Shahabi, M. A. Shaikh, M. Shannawaz, M. S. R. Shawon, A. Sheikh, S. Sheikhbahaei, K. Shibuya, S. Siabani, D. A. S. Silva, J. A. Singh, J. K. Singh, V. Y. Skryabin, A. A. Skryabina, B. H. Sobaih, S. Stortecky, S. Stranges, E. G. Tadesse, I. U. Tarigan, M. H. Temsah, Y. Teuschl, A. G. Thrift, M. Tonelli, M. R. Tovani-Palone, B. X. Tran, M. Tripathi, G. W. Tsegaye, A. Ullah, B. Unim, B. Unnikrishnan, A. Vakilian, S. Valadan Tahbaz, T. J. Vasankari, N. Venketasubramanian, D. Vervoort, B. Vo, V. Volovici, K. Vosoughi, G. T. Vu, L. G. Vu, H. A. Wafa, Y. Waheed, Y. Wang, T. Wijeratne, A. S. Winkler, C. D. A. Wolfe, M. Woodward, J. H. Wu, S. Wulf Hanson, X. Xu, L. Yadav, A. Yadollahpour, S. H. Yahyazadeh Jabbari, K. Yamagishi, H. Yatsuya, N. Yonemoto, C. Yu, I. Yunusa, M. S. Zaman, S. B. Zaman, M. Zamanian, R. Zand, A. Zandifar, M. S. Zastrozhin, A. Zastrozhina, Y. Zhang, Z. J. Zhang, C. Zhong, Y. M. H. Zuniga, C. J. L. Murray, Global, regional, and national burden of stroke and its risk factors, 1990-2019: A systematic analysis for the Global Burden of Disease Study 2019. Lancet Neurol. 20, 795–820 (2021). 10.1016/S1474-4422(21)00252-0 PMC8443449 34487721

[2] GBD 2016 Lifetime Risk of Stroke Collaborators, Global, regional, and country-specific lifetime risks of stroke, 1990 and 2016. N. Engl. J. Med. 379, 2429–2437 (2018). 10.1056/NEJMoa1804492 PMC6247346 30575491

[3] F. Akbik, H. Xu, Y. Xian, S. Shah, E. E. Smith, D. L. Bhatt, R. A. Matsouaka, G. C. Fonarow, L. H. Schwamm, Trends in reperfusion therapy for in-hospital ischemic stroke in the endovascular therapy era. JAMA Neurol. 77, 1486–1495 (2020). 10.1001/jamaneurol.2020.3362 PMC7506601 32955582

[4] A. W. Dromerick, S. Geed, J. Barth, K. Brady, M. L. Giannetti, A. Mitchell, M. A. Edwardson, M. T. Tan, Y. Zhou, E. L. Newport, D. F. Edwards, Critical Period After Stroke Study (CPASS): A phase II clinical trial testing an optimal time for motor recovery after stroke in humans. Proc. Natl. Acad. Sci. U.S.A. 118, e2026676118 (2021). 10.1073/pnas.2026676118 PMC8488696 34544853

[5] T. H. Murphy, D. Corbett, Plasticity during stroke recovery: From synapse to behaviour. Nat. Rev. Neurosci. 10, 861–872 (2009). 10.1038/nrn2735 19888284

[6] D. B. Chklovskii, B. W. Mel, K. Svoboda, Cortical rewiring and information storage. Nature 431, 782–788 (2004). 10.1038/nature03012 15483599

[7] A. Holtmaat, P. Caroni, Functional and structural underpinnings of neuronal assembly formation in learning. Nat. Neurosci. 19, 1553–1562 (2016). 10.1038/nn.4418 27749830

[8] T. F. Roberts, K. A. Tschida, M. E. Klein, R. Mooney, Rapid spine stabilization and synaptic enhancement at the onset of behavioural learning. Nature 463, 948–952 (2010). 10.1038/nature08759 PMC2918377 20164928

[9] A. C. Frank, S. Huang, M. Zhou, A. Gdalyahu, G. Kastellakis, T. K. Silva, E. Lu, X. Wen, P. Poirazi, J. T. Trachtenberg, A. J. Silva, Hotspots of dendritic spine turnover facilitate clustered spine addition and learning and memory. Nat. Commun. 9, 422 (2018). 10.1038/s41467-017-02751-2 PMC5789055 29379017

[10] A. Hayashi-Takagi, S. Yagishita, M. Nakamura, F. Shirai, Y. I. Wu, A. L. Loshbaugh, B. Kuhlman, K. M. Hahn, H. Kasai, Labelling and optical erasure of synaptic memory traces in the motor cortex. Nature 525, 333–338 (2015). 10.1038/nature15257 PMC4634641 26352471

[11] M. T. Joy, S. T. Carmichael, Encouraging an excitable brain state: Mechanisms of brain repair in stroke. Nat. Rev. Neurosci. 22, 38–53 (2021). 10.1038/s41583-020-00396-7 PMC10625167 33184469

[12] C. E. Brown, P. Li, J. D. Boyd, K. R. Delaney, T. H. Murphy, Extensive turnover of dendritic spines and vascular remodeling in cortical tissues recovering from stroke. J. Neurosci. 27, 4101–4109 (2007). 10.1523/JNEUROSCI.4295-06.2007 PMC6672555 17428988

[13] C. E. Brown, K. Aminoltejari, H. Erb, I. R. Winship, T. H. Murphy, In vivo voltage-sensitive dye imaging in adult mice reveals that somatosensory maps lost to stroke are replaced over weeks by new structural and functional circuits with prolonged modes of activation within both the peri-infarct zone and distant sites. J. Neurosci. 29, 1719–1734 (2009). 10.1523/JNEUROSCI.4249-08.2009 PMC6666293 19211879

[14] L. Wang, J. M. Conner, A. H. Nagahara, M. H. Tuszynski, Rehabilitation drives enhancement of neuronal structure in functionally relevant neuronal subsets. Proc. Natl. Acad. Sci. U.S.A. 113, 2750–2755 (2016). 10.1073/pnas.1514682113 PMC4790976 26903653

[15] T. A. Clark, C. Sullender, D. Jacob, Y. Zuo, A. K. Dunn, T. A. Jones, Rehabilitative training interacts with ischemia-instigated spine dynamics to promote a lasting population of new synapses in peri-infarct motor cortex. J. Neurosci. 39, 8471–8483 (2019). 10.1523/JNEUROSCI.1141-19.2019 PMC6807284 31511430

[16] S. Ge, J. A. Dani, Nicotinic acetylcholine receptors at glutamate synapses facilitate long-term depression or potentiation. J. Neurosci. 25, 6084–6091 (2005). 10.1523/JNEUROSCI.0542-05.2005 PMC6725070 15987938

[17] Z. Gu, J. L. Yakel, Timing-dependent septal cholinergic induction of dynamic hippocampal synaptic plasticity. Neuron 71, 155–165 (2011). 10.1016/j.neuron.2011.04.026 PMC3134790 21745645

[18] T. D. Farr, I. Q. Whishaw, Quantitative and qualitative impairments in skilled reaching in the mouse (Mus musculus) after a focal motor cortex stroke. Stroke 33, 1869–1875 (2002). 10.1161/01.str.0000020714.48349.4e 12105368

[19] J. Biernaskie, G. Chernenko, D. Corbett, Efficacy of rehabilitative experience declines with time after focal ischemic brain injury. J. Neurosci. 24, 1245–1254 (2004). 10.1523/JNEUROSCI.3834-03.2004 PMC6793570 14762143

[20] J. M. Miwa, I. Ibanez-Tallon, G. W. Crabtree, R. Sánchez, A. Sali, L. W. Role, N. Heintz, lynx1, an endogenous toxin-like modulator of nicotinic acetylcholine receptors in the mammalian CNS. Neuron 23, 105–114 (1999). 10.1016/s0896-6273(00)80757-6 10402197

[21] J. M. Miwa, Lynx1 prototoxins: Critical accessory proteins of neuronal nicotinic acetylcholine receptors. Curr. Opin. Pharmacol. 56, 46–51 (2021). 10.1016/j.coph.2020.09.016 PMC8771676 33254061

[22] H. Morishita, J. M. Miwa, N. Heintz, T. K. Hensch, Lynx1, a cholinergic brake, limits plasticity in adult visual cortex. Science 330, 1238–1240 (2010). 10.1126/science.1195320 PMC3387538 21071629

[23] M. Sajo, G. Ellis-Davies, H. Morishita, Lynx1 limits dendritic spine turnover in the adult visual cortex. J. Neurosci. 36, 9472–9478 (2016). 10.1523/JNEUROSCI.0580-16.2016 PMC5013192 27605620

[24] E. N. Falk, K. J. Norman, Y. Garkun, M. P. Demars, S. Im, G. Taccheri, J. Short, K. Caro, S. E. McCraney, C. Cho, M. R. Smith, H.-M. Lin, H. Koike, J. Bateh, P. Maccario, L. Waltrip, M. Janis, H. Morishita, Nicotinic regulation of local and long-range input balance drives top-down attentional circuit maturation. Sci. Adv. 7, eabe1527 (2021). 10.1126/sciadv.abe1527 PMC7935362 33674307

[25] Z. Gan, V. Gangadharan, S. Liu, C. Körber, L. L. Tan, H. Li, M. J. Oswald, J. Kang, J. Martin-Cortecero, D. Männich, A. Groh, T. Kuner, S. Wieland, R. Kuner, Layer-specific pain relief pathways originating from primary motor cortex. Science 378, 1336–1343 (2022). 10.1126/science.add4391 36548429

[26] T. Xu, X. Yu, A. J. Perlik, W. F. Tobin, J. A. Zweig, K. Tennant, T. Jones, Y. Zuo, Rapid formation and selective stabilization of synapses for enduring motor memories. Nature 462, 915–919 (2009). 10.1038/nature08389 PMC2844762 19946267

[27] G. Yang, F. Pan, W.-B. Gan, Stably maintained dendritic spines are associated with lifelong memories. Nature 462, 920–924 (2009). 10.1038/nature08577 PMC4724802 19946265

[28] M. Fu, X. Yu, J. Lu, Y. Zuo, Repetitive motor learning induces coordinated formation of clustered dendritic spines in vivo. Nature 483, 92–95 (2012). 10.1038/nature10844 PMC3292711 22343892

[29] R. H. Roth, R. H. Cudmore, H. L. Tan, I. Hong, Y. Zhang, R. L. Huganir, Cortical synaptic AMPA receptor plasticity during motor learning. Neuron 105, 895–908.e5 (2020). 10.1016/j.neuron.2019.12.005 PMC7060107 31901303

[30] E. Albarran, A. Raissi, O. Jáidar, C. J. Shatz, J. B. Ding, Enhancing motor learning by increasing the stability of newly formed dendritic spines in the motor cortex. Neuron 109, 3298–3311.e4 (2021). 10.1016/j.neuron.2021.07.030 PMC8542616 34437845

[31] N. G. Hedrick, W. J. Wright, T. Komiyama, Local and global predictors of synapse elimination during motor learning. Sci. Adv. 10, eadk0540 (2024). 10.1126/sciadv.adk0540 PMC10942101 38489360

[32] K. A. Tennant, S. L. Taylor, E. R. White, C. E. Brown, Optogenetic rewiring of thalamocortical circuits to restore function in the stroke injured brain. Nat. Commun. 8, 15879 (2017). 10.1038/ncomms15879 PMC5490053 28643802

[33] I. Ibañez-Tallon, J. M. Miwa, H. L. Wang, N. C. Adams, G. W. Crabtree, S. M. Sine, N. Heintz, Novel modulation of neuronal nicotinic acetylcholine receptors by association with the endogenous prototoxin lynx1. Neuron 33, 893–903 (2002). 10.1016/s0896-6273(02)00632-3 11906696

[34] J. M. Miwa, T. R. Stevens, S. L. King, B. J. Caldarone, I. Ibanez-Tallon, C. Xiao, R. M. Fitzsimonds, C. Pavlides, H. A. Lester, M. R. Picciotto, N. Heintz, The prototoxin lynx1 acts on nicotinic acetylcholine receptors to balance neuronal activity and survival in vivo. Neuron 51, 587–600 (2006). 10.1016/j.neuron.2006.07.025 16950157

[35] C. Dong, N. R. Kern, K. R. Anderson, X. F. Zhang, J. M. Miwa, W. Im, Dynamics and interactions of GPI-linked lynx1 protein with/without nicotinic acetylcholine receptor in membrane bilayers. J. Phys. Chem. B 124, 4017–4025 (2020). 10.1021/acs.jpcb.0c00159 PMC7820712 32208709

[36] W. A. Nichols, B. J. Henderson, C. Yu, R. L. Parker, C. I. Richards, H. A. Lester, J. M. Miwa, Lynx1 shifts α4β2 nicotinic receptor subunit stoichiometry by affecting assembly in the endoplasmic reticulum. J. Biol. Chem. 289, 31423–31432 (2014). 10.1074/jbc.M114.573667 PMC4223341 25193667

[37] Y. Nakahata, R. Yasuda, Plasticity of spine structure: Local signaling, translation and cytoskeletal reorganization. Front. Synaptic Neurosci. 10, 29 (2018). 10.3389/fnsyn.2018.00029 PMC6123351 30210329

[38] A. F. Lozada, X. Wang, N. V. Gounko, K. A. Massey, J. Duan, Z. Liu, D. K. Berg, Induction of dendritic spines by β2-containing nicotinic receptors. J. Neurosci. 32, 8391–8400 (2012). 10.1523/JNEUROSCI.6247-11.2012 PMC3387687 22699919

[39] B. J. Morley, R. F. Mervis, Dendritic spine alterations in the hippocampus and parietal cortex of alpha7 nicotinic acetylcholine receptor knockout mice. Neuroscience 233, 54–63 (2013). 10.1016/j.neuroscience.2012.12.025 PMC3658285 23270857

[40] M. Matsuzaki, N. Honkura, G. C. Ellis-Davies, H. Kasai, Structural basis of long-term potentiation in single dendritic spines. Nature 429, 761–766 (2004). 10.1038/nature02617 PMC4158816 15190253

[41] Q. Zhou, K. J. Homma, M.-M. Poo, Shrinkage of dendritic spines associated with long-term depression of hippocampal synapses. Neuron 44, 749–757 (2004). 10.1016/j.neuron.2004.11.011 15572107

[42] C. Lee, B. K. Kaang, Clustering of synaptic engram: Functional and structural basis of memory. Neurobiol. Learn. Mem. 216, 107993 (2024). 10.1016/j.nlm.2024.107993 39424222

[43] M. R. Picciotto, M. J. Higley, Y. S. Mineur, Acetylcholine as a neuromodulator: Cholinergic signaling shapes nervous system function and behavior. Neuron 76, 116–129 (2012). 10.1016/j.neuron.2012.08.036 PMC3466476 23040810

[44] M. R. Ananth, P. Rajebhosale, R. Kim, D. A. Talmage, L. W. Role, Basal forebrain cholinergic signalling: Development, connectivity and roles in cognition. Nat. Rev. Neurosci. 24, 233–251 (2023). 10.1038/s41583-023-00677-x PMC10439770 36823458

[45] B. Hangya, S. P. Ranade, M. Lorenc, A. Kepecs, Central cholinergic neurons are rapidly recruited by reinforcement feedback. Cell 162, 1155–1168 (2015). 10.1016/j.cell.2015.07.057 PMC4833212 26317475

[46] L. M. Teles-Grilo Ruivo, K. L. Baker, M. W. Conway, P. J. Kinsley, G. Gilmour, K. G. Phillips, J. T. R. Isaac, J. P. Lowry, J. R. Mellor, Coordinated acetylcholine release in prefrontal cortex and hippocampus is associated with arousal and reward on distinct timescales. Cell Rep. 18, 905–917 (2017). 10.1016/j.celrep.2016.12.085 PMC5289927 28122241

[47] S. Bowles, J. Hickman, X. Peng, W. R. Williamson, R. Huang, K. Washington, D. Donegan, C. G. Welle, Vagus nerve stimulation drives selective circuit modulation through cholinergic reinforcement. Neuron 110, 2867–2885.e7 (2022). 10.1016/j.neuron.2022.06.017 PMC10212211 35858623

[48] T. Nagata, C. A. Dwyer, K. Yoshida-Tanaka, K. Ihara, M. Ohyagi, H. Kaburagi, H. Miyata, S. Ebihara, K. Yoshioka, T. Ishii, K. Miyata, K. Miyata, B. Powers, T. Igari, S. Yamamoto, N. Arimura, H. Hirabayashi, T. Uchihara, R. I. Hara, T. Wada, C. F. Bennett, P. P. Seth, F. Rigo, T. Yokota, Cholesterol-functionalized DNA/RNA heteroduplexes cross the blood-brain barrier and knock down genes in the rodent CNS. Nat. Biotechnol. 39, 1529–1536 (2021). 10.1038/s41587-021-00972-x 34385691

[49] S. J. Barker, M. B. Thayer, C. Kim, D. Tatarakis, M. J. Simon, R. Dial, L. Nilewski, R. C. Wells, Y. Zhou, M. Afetian, P. Akkapeddi, A. Chappell, K. S. Chew, J. Chow, A. Clemens, C. B. Discenza, J. C. Dugas, C. Dwyer, T. Earr, C. Ha, Y. S. Ho, D. Huynh, E. I. Lozano, S. Jayaraman, W. Kwan, C. Mahon, M. Pizzo, Y. Robles-Colmenares, E. Roche, L. Sanders, A. Stergioulis, R. Tong, H. Tran, Y. Zuchero, A. A. Estrada, K. Gadkar, C. M. M. Koth, P. E. Sanchez, R. G. Thorne, R. J. Watts, T. Sandmann, L. A. Kane, F. Rigo, M. S. Dennis, J. W. Lewcock, S. L. DeVos, Targeting the transferrin receptor to transport antisense oligonucleotides across the mammalian blood-brain barrier. Sci. Transl. Med. 16, eadi2245 (2024). 10.1126/scitranslmed.adi2245 39141703

[50] G. Feng, R. H. Mellor, M. Bernstein, C. Keller-Peck, Q. T. Nguyen, M. Wallace, J. M. Nerbonne, J. W. Lichtman, J. R. Sanes, Imaging neuronal subsets in transgenic mice expressing multiple spectral variants of GFP. Neuron 28, 41–51 (2000). 10.1016/s0896-6273(00)00084-2 11086982

[51] A. Holtmaat, T. Bonhoeffer, D. K. Chow, J. Chuckowree, V. De Paola, S. B. Hofer, M. Hübener, T. Keck, G. Knott, W.-C. A. Lee, R. Mostany, T. D. Mrsic-Flogel, E. Nedivi, C. Portera-Cailliau, K. Svoboda, J. T. Trachtenberg, L. Wilbrecht, Long-term, high-resolution imaging in the mouse neocortex through a chronic cranial window. Nat. Protoc. 4, 1128–1144 (2009). 10.1038/nprot.2009.89 PMC3072839 19617885

[52] C. C. Chen, A. Gilmore, Y. Zuo, Study motor skill learning by single-pellet reaching tasks in mice. J. Vis. Exp., e51238 (2014). 10.3791/51238 PMC4123783 24637358

[53] Y. Li, E. R. Hollis II, Nicotinic acetylcholine signaling is required for motor learning but not for rehabilitation from spinal cord injury. Neural Regen. Res. 18, 364–367 (2023). 10.4103/1673-5374.346544 PMC9396487 35900431

[54] L. Wieczorek, J. W. Maas Jr., L. M. Muglia, S. K. Vogt, L. J. Muglia, Temporal and regional regulation of gene expression by calcium-stimulated adenylyl cyclase activity during fear memory. PLOS ONE 5, e13385 (2010). 10.1371/journal.pone.0013385 PMC2954788 20976279

[55] Y. Hiraoka, K. Yamada, Y. Kawasaki, H. Hirose, Y. Matsumoto, K. Ishikawa, Y. Yasumizu, ikra: RNAseq pipeline centered on Salmon. (Zenodo, 2019); 10.5281/zenodo.3352573.

[56] S. X. Ge, E. W. Son, R. Yao, iDEP: An integrated web application for differential expression and pathway analysis of RNA-Seq data. BMC Bioinformatics 19, 534 (2018). 10.1186/s12859-018-2486-6 PMC6299935 30567491

[57] Q. Wang, S.-L. Ding, Y. Li, J. Royall, D. Feng, P. Lesnar, N. Graddis, M. Naeemi, B. Facer, A. Ho, T. Dolbeare, B. Blanchard, N. Dee, W. Wakeman, K. E. Hirokawa, A. Szafer, S. M. Sunkin, S. W. Oh, A. Bernard, J. W. Phillips, M. Hawrylycz, C. Koch, H. Zeng, J. A. Harris, L. Ng, The Allen Mouse Brain Common Coordinate Framework: A 3D reference atlas. Cell 181, 936–953.e20 (2020). 10.1016/j.cell.2020.04.007 PMC8152789 32386544

[58] K. A. Tennant, D. L. Adkins, N. A. Donlan, A. L. Asay, N. Thomas, J. A. Kleim, T. A. Jones, The organization of the forelimb representation of the C57BL/6 mouse motor cortex as defined by intracortical microstimulation and cytoarchitecture. Cereb. Cortex 21, 865–876 (2011). 10.1093/cercor/bhq159 PMC3059888 20739477

