# Supplementary material for: Lynx1 suppression reopens the window for effective rehabilitation after stroke in mice
Source: Sci Adv. 2026 Nov 13;12(46):eaed9140. doi: 10.1126/sciadv.aed9140 (PMC13763986; doi:10.1126/sciadv.aed9140)
Supplement: Supplementary file 1 [file sciadv.aed9140_sm.pdf]

Science Advances
AAAS

Supplementary Materials for

Lynx1 suppression reopens the window for effective rehabilitation after stroke in mice

Toru Nakanishi *et al.*

Corresponding author: Takahide Itokazu, t.itokazu@molneu.med.osaka-u.ac.jp;
Toshihide Yamashita, yamashita@molneu.med.osaka-u.ac.jp

Sci. Adv. **12**, eaed9140 (2026)
DOI: 10.1126/sciadv.aed9140

This PDF file includes:

Fig. S1

Figure S1.

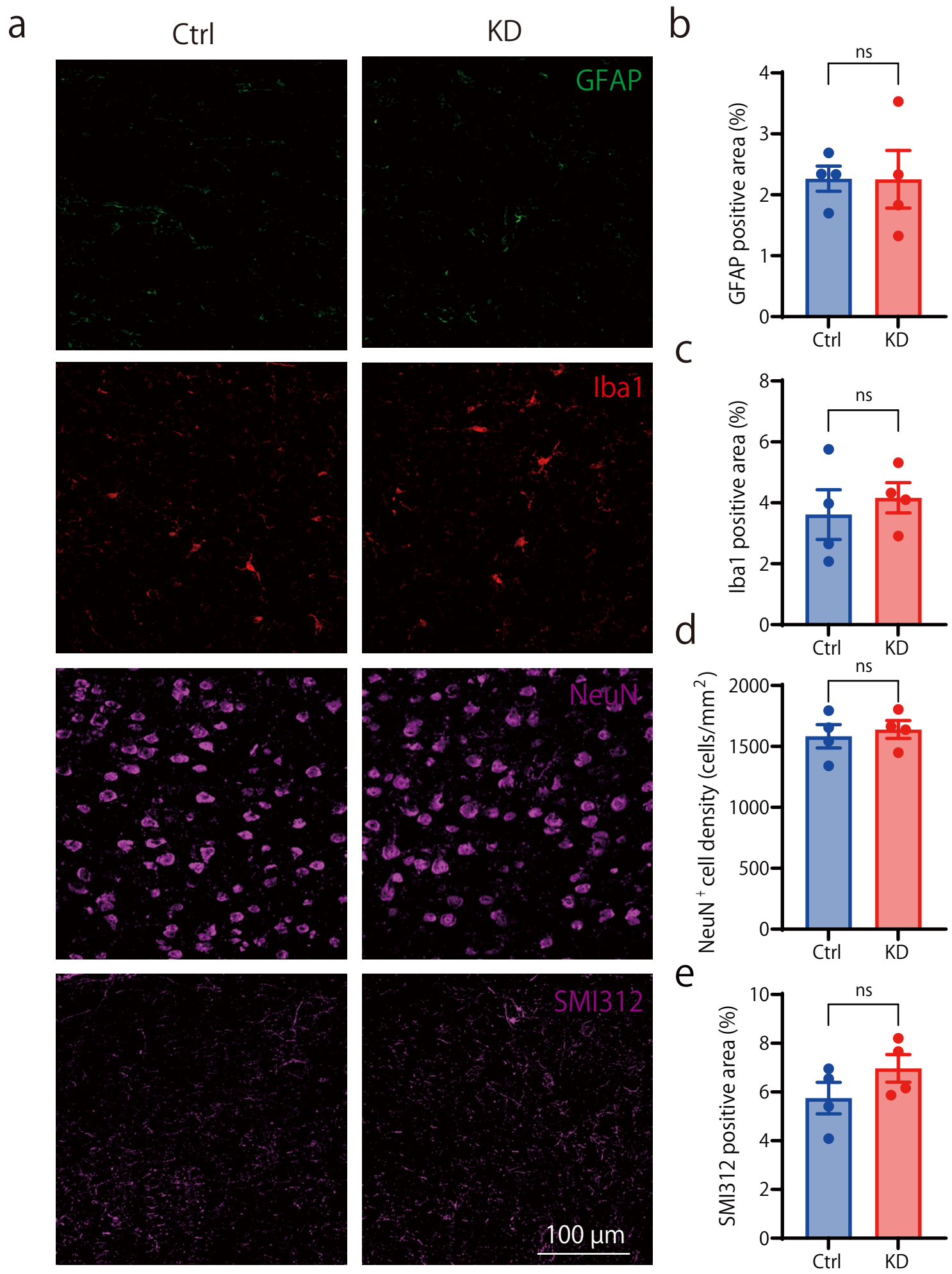
a
Ctrl
KD
GFAP
Iba1
NeuN
SMI312
100 µm
b
ns
GFAP positive area (%)
Ctrl
KD
c
ns
Iba1 positive area (%)
d
ns
NeuN⁺ cell density (cells/mm²)
e
ns
SMI312 positive area (%)

Figure S1. Lynx1 knockdown does not alter the histological characteristics of the peri-infarct area.

(**a**) Representative images of GFAP, Iba1, NeuN, and SMI312 immunostaining in the peri-infarct area of mice treated with control ASO (Ctrl) or Lynx1 ASO (KD). Scale bar: 100 µm.

(**b–e**) Quantification of the GFAP-positive area (b), Iba1-positive area (c), NeuN-positive cell density (d), and SMI312-positive area (e) in the peri-infarct area (n = 3). Data are presented as mean ± SEM. ns, not significant; Student's *t*-test.
